# Do neighborhood demographics, crime rates, and alcohol outlet density predict incidence, severity, and outcome of hospitalization for traumatic injury? A cross-sectional study of Dallas County, Texas, 2010

**DOI:** 10.1186/s40621-014-0023-2

**Published:** 2014-10-20

**Authors:** Alan Cook, Jennifer Reingle Gonzalez, Bijal A Balasubramanian

**Affiliations:** 1 Chandler Regional Medical Center, 485 South Dobson Road, Suite 201, Chandler, 85224 AZ USA; 2 University of Texas School of Public Health, Dallas Regional Campus, Dallas, 75390 Texas USA

**Keywords:** Socioeconomic status, Crime, Trauma, Injury, Mortality, Alcohol outlets

## Abstract

**Background:**

Unintentional injury leads all other causes of death for those 1 to 45 years old. The expense of medical care for injured people is estimated to exceed $406 billion annually. Given this burden on the population, the Centers for Disease Control and Prevention consistently refers to injury prevention as a national priority. We postulated that exposure to crime and the density of alcohol outlets in one's neighborhood will be positively associated with the incidence of hospitalization for and mortality from traumatic injuries, independent of other neighborhood characteristics.

**Methods:**

We conducted a cross-sectional study with ecological and individual analyses. Patient-level data for traumatic injury, injury severity, and hospital mortality due to traumatic injury in 2010 were gathered from the Dallas-Fort Worth Hospital Council Foundation. Each case of traumatic injury or death was geospatially linked with neighborhood of origin information from the 2010 U.S. Census within Dallas County, Texas. This information was subsequently linked with crime data gathered from 20 local police departments and the Texas Alcoholic Beverage Commission alcohol outlet dataset. The crime data are the Part One crimes reported to the Federal Bureau of Investigation.

**Results:**

The proportion of persons 65 years old or older was the strongest predictor of the incidence of hospitalization for traumatic injury (*b =* 12.64, 95% confidence interval (CI) 8.73 to 16.55). In turn, the incidence of traumatic injury most strongly predicted the severity of traumatic injury (*b =* 0.008, 95% CI 0.0003 – 0.0012). The tract-level unemployment rate was associated with a 5% increase in the odds of hospital mortality among hospitalized trauma patients.

**Conclusions:**

Several neighborhood characteristics were associated with the incidence, severity, and hospital mortality from traumatic injury. However, crime rates and alcohol outlet density carried no such association. Prevention efforts should focus on neighborhood characteristics such as population density, mean age of the residents, and unemployment rate, regardless of crime rates and alcohol outlet density.

**Electronic supplementary material:**

The online version of this article (doi:10.1186/s40621-014-0023-2) contains supplementary material, which is available to authorized users.

## 1 Background

Traumatic injuries cost more than $406 billion in medical care and lost productivity annually (Finkelstein et al. [2006]), and unintentional injury remains the leading cause of death among several age groups in the USA (Centers for Disease Control and Prevention [2010]). Thus, injury prevention is a national priority. Across numerous medical disciplines, socioeconomic status (SES) by various measures has been strongly associated with higher incidence rates, and severity of disease including cancers and cardiovascular diseases (Kucharska-Newton et al. [2011]; Schwartz et al. [2003]), greater operative mortality (Bennett et al. [2010]), and poorer overall survival rates represented disproportionately in lower SES populations (Daly et al. [2002]). Previous research suggest that neighborhood characteristics, including measures of SES, may reflect the relative availability and access to resources necessary for well-being which can in turn influence outcomes of medical care, particularly, in light of the limited access to healthcare in low-income communities (Braveman et al. [1988]).

Because SES is a multidimensional construct (that continues to generate definitional debate), it is particularly important that multiple measurement sources be investigated to elucidate the effect of ‘SES’ along with other neighborhood characteristics on trauma incidence, injury severity, and hospital mortality. Previously identified predictors include the following: individual income, employment status, and education level (individual level) and household income, mean or median education level, and proportion of a population living below a given poverty level (neighborhood level) (Galobardes et al. [2006a], [b]; Daly et al. [2002]). Prior studies characterizing traumatic injury within social contexts often focus on singular facets of a complex mosaic of economic and social neighborhood attributes (Newgard et al. [2011]); however, each study has its own respective limitations. Specifically, the exclusion of one or more important confounding measures may distort the relationship between SES and the phenomena of traumatic injury (Cubbin et al. [2000]; Hefernan et al. [2011]; Marcin et al. [2003]; Newgard et al. [2011]; Zarzur et al. [2010]; Rosen et al. [2009]). As such, other factors shown to be associated with the occurrence and outcomes of traumatic injury must be taken into account.

Alcohol retail outlet density is one such factor. The associations between alcohol retail outlet density and pedestrian injury collisions, car crashes and related injuries, and assaults, including intimate partner violence have all been well described (LaScala et al. [2001]; Treno et al. [2007]; Holder et al. [2000]; Scribner et al. [1995]). Alcohol retail outlet density has been found to be associated with various neighborhood characteristics, including poverty and racial demographics (Berke et al. [2010]). Each additional alcohol outlet has been shown to increase crime reports to police by 3%. Moreover, alcohol has long been linked to the commission of crimes (Shupe [1953]; Ladouceur and Temple [1985]). Finally, both interpersonal and property crimes are associated with the incidence of trauma (Hashima and Finkelhor [1999]; Zimring and Zuehl [1986]; Wilkinson et al. [2008]; Cook [1986]).

Clearly, a deeper understanding of the complex interactions between environmental contexts, individual behavior, exposure to crime, and traumatic injury may inform and improve injury prevention initiatives. We conducted an epidemiologic investigation that comprehensively examined the relationship between multidimensional indicators of SES with a focus on crime rates and alcohol retail density in the neighborhood as risk factors for the incidence and severity of, and mortality from, traumatic injury, in Dallas County, Texas. This study is particularly unique in that contextual measures were geographically linked from larger data sources (e.g., the U.S. Census) via geocoding of trauma patient home addresses. We postulated that exposure to crime and the density of alcohol outlets in one's neighborhood will be positively associated with the incidence of hospitalization for and mortality from traumatic injuries, independent of established SES measures such as income, employment status, and education. Therefore, we sought to answer two specific research questions. First, are the crime rate and alcohol retail density in a patients' census tract positively associated with their odds of hospitalization for traumatic injury? Second, do crime rates and alcohol retail density positively predict higher levels of injury severity and mortality, independent of other neighborhood contextual measures, patient behavior, and demographic measures? These two research questions are systematically evaluated in this study.

## 2 Methods

### 2.1 Data sources

#### 2.1.1 Contextual U.S. Census, crime, and alcohol outlet data

SES data were obtained from the 2010 United States Census website (U. S. Census Bureau [2012]). These data included population estimates for each census tract included in this study, age and sex distributions, education levels, proportions of persons living below the federal poverty level, median household income, employment rates, housing information (such as rates of owner-occupied housing), and race/ethnicity information. Crime, alcohol retail establishment, and patients' hospitalization data were restricted to the year 2010 to be consistent with the United States Census data.

Data for major crimes were obtained via the Uniform Crime Reports (UCR) Part One crimes (Federal Bureau of Investigation [2004]) which include homicide, forcible rape, robbery, assault, burglary (breaking and entering), larceny/theft (including theft from motor vehicles), unauthorized use of motor vehicles (theft of the motor vehicle), and arson. Crime data for 2010, including the type of crime and the address where the crime occurred was provided by 20 of the 26 cities in Dallas County. Patients from the six cities that did not provide data to the UCR were excluded from the study. Finally, the Texas Alcoholic Beverage Commission provided the addresses of alcoholic beverage retail establishments throughout the area of interest in this study. This allowed us to compute a ‘density’ of alcohol outlets given the estimated population size of each patient's ‘neighborhood’ census tract (alcohol retail outlets per census tract/estimated population size per census tract × 100,000 = alcohol retail outlets per 100,000 population for a given census tract) (Berke et al. [2010]). We included only those alcohol retailers with active licenses during 2010.

**Figure 1:**
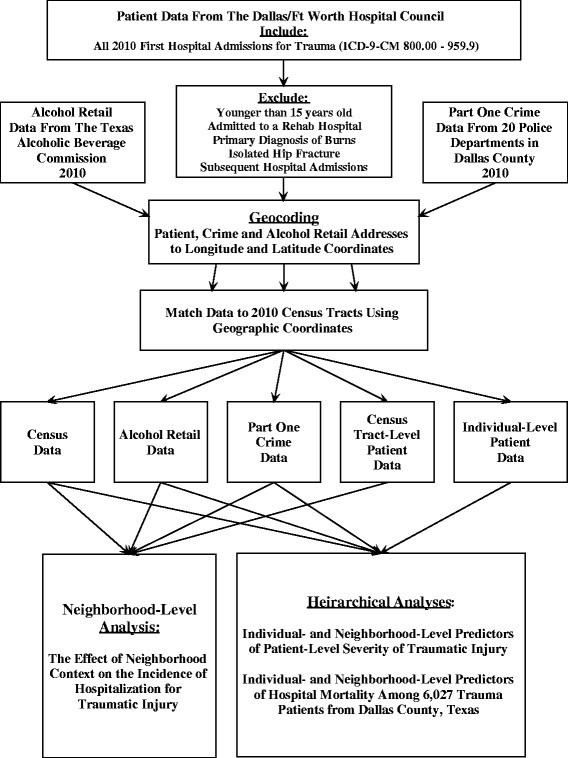
**Process of acquiring, matching, and analyzing data.**

Patients were included in this study if they lived within 1 of the 20 police jurisdictions in Dallas County, Texas that provided crime data to the Federal Bureau of Investigation's Uniform Crime Reports (UCR) (Figure 1). The patients' initial hospitalization for traumatic injuries occurred during 2010. Any subsequent hospital admission for any individual patient was excluded from the dataset. Patients were excluded if they were 14 years old or younger or hospitalized for burns as the primary diagnosis, as these groups have their own injury and mortality prediction models.

#### 2.1.2 Patients: individual-level data

Patient-level data were collected directly from the Dallas-Fort Worth Hospital Council Foundation (DFWHCF), an organization that stores data from more than 90% of all hospital discharges across the North Texas region. After receiving institutional review board approval, we obtained patient-level data for each discharge, including self-reported patient demographics, home address, type of insurance, length of stay, International Classification of Diseases, Ninth Edition - Clinical Modification (ICD-9) codes for diagnoses, procedures, and external causes of injury and poisoning (E-codes). Comorbid conditions were enumerated using the Elixhauser Comorbidity Score (Elixhauser et al. [1998]).

#### 2.1.3 Outcomes: trauma-related morbidity and mortality

The severity of traumatic injury was measured as the probability of mortality using the Trauma Mortality Prediction Model for the ICD-9 lexicon (Glance et al. [2009]). Hospital discharge status identified each patient as either alive or deceased.

### 2.2 Statistical analysis

#### 2.2.1 Geocoding and data linkage

The addresses of patients hospitalized for traumatic injury were linked to crime rates and alcohol retail densities in their respective census tracts using ArcGIS version 10.1 (ESRI, Redlands, CA, USA) and the user-written Geocode routine for Stata 12 (StataCorp., College Station, TX, USA). The geocoding process was a point-in-polygon method using TIGER/Line shapefiles provided online by the U. S. Census Bureau ([2012]). All crime rates and alcohol retail densities were population-adjusted/100,000 persons per census tract.

Multi-level predictors of injury severity and hospital mortality were assessed using hierarchical regression methods in Stata 12 (StataCorp., College Station, TX, USA). Predictor variables were excluded from multivariate analysis if they were not related to the outcome in *any* bivariate analyses, unless they were theoretically related to our research questions (e.g., represented crime rates or alcohol outlet density), or were identified as particularly relevant predictors of traumatic injury in the literature reviewed above.

#### 2.2.2 Multi-level regression analyses

**Figure 2:**
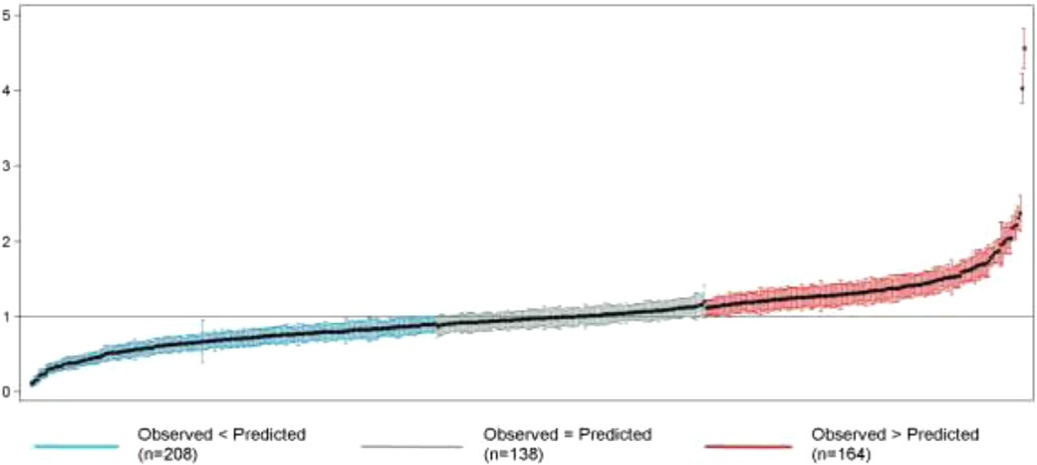
**Standardized incidence ratios for traumas per 100,000 populations in 510 census tracts, Dallas County.**

**Figure 3:**
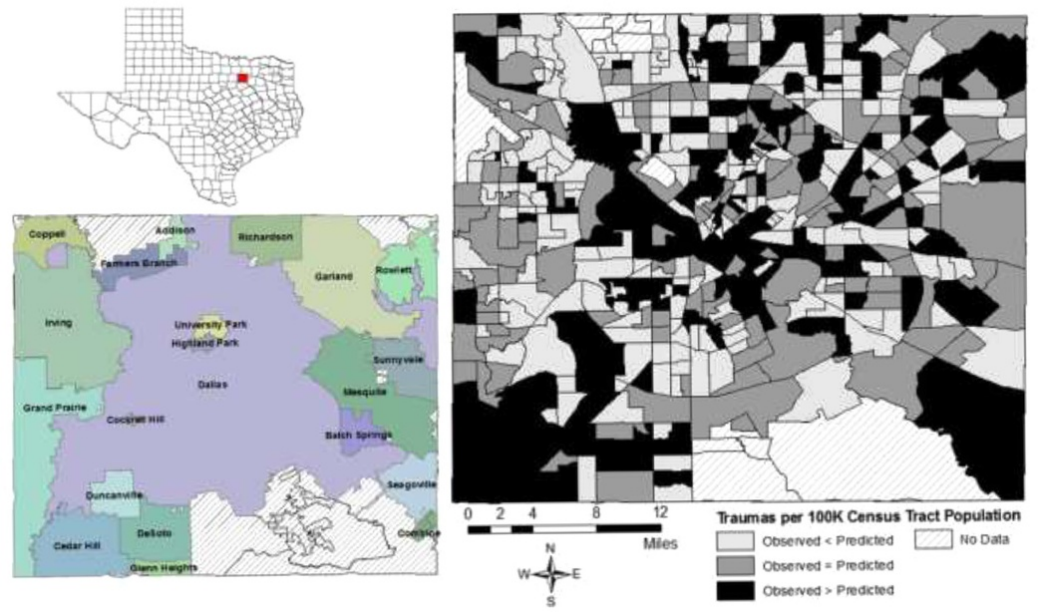
**Standardized incidence rates of traumas per 100,000 census tract population, Dallas County.**

Patient-level hospital mortality was analyzed using a hierarchical logit model (Moore et al. [2010]) which corrected for random effects at both the hospital and census tract levels. Model discrimination was reported as area under the receiver operator characteristic (ROC) curve (Hosmer Jr et al. [2013]). The variation in trauma/100,000 population among census tracts was analyzed as standardized incidence ratios (SIR), and for clarity, these results are presented as tables and geographically below (Figures 2 and 3). Sensitivity analyses were conducted to ensure that the six jurisdictions excluded from this study (because crime data were not available) did not differ from those included in the analysis on any relevant outcome or predictor variable.

## 3 Results

According to the 2010 U.S. Census, the population of Dallas County was estimated to be 2,348,702 people, residing within 526 census tracts and occupying 908 square miles. The geographic boundary of this study encompassed 510 contiguous census tracts covering 89% of the total land area in Dallas County. Due to missing crime data by jurisdiction, the total population denominator for this study was 2,279,737, which represented 97.1% of the county population. Using data from the DFWHCF, 6,032 trauma patients met our selection criteria for inclusion. The descriptive characteristics of the sample, census tracts, and all independent and dependent variables at the individual and census tract levels are detailed in Table 1. There were 17,550 injuries, representing 1,040 separate ICD-9 codes, diagnosed in our cohort of 6,032 patients. A breakdown of these ICD-9 codes is provided in Tables 2, 3, and 4.

**Table 1:** **Descriptive profile of individual and multi-level measures included in this analysis**

	***n*** (%)	Mean (95% CI) or median (range)+
Outcomes		
Mortality (discharged as ‘dead’)	151 (3.5)	
Hospitalizations for traumatic injury/100,000	-	Median, 263.46; range, 21.15 to 1,560.14
Trauma patients/census tract	-	Median, 11; range, 1 to 58
Trauma Mortality Prediction Model p(death)	-	Median, 1.57%; range, 0.001% to 94.01%
Independent variables: contextual		
Census measures: socioeconomic indicators		
% Owner-occupied housing	-	54.3 (52.0 to 56.7)
% of Population less than 18 years old	-	26.4 (25.7 to 27.2)
% Estimated living below poverty level	-	18.7 (17.6 to 19.9)
% Unemployed (previous 12 months)	-	7.7 (7.3 to 8.1)
% High school graduates	-	75.8 (74.3 to 77.4)
Median annual household ($)	-	Median, $46,188; range, $11,875 to $247,292
Alcohol outlets/100,000 population	-	49.8 (41.9 to 57.7)
Dallas county UCR crime rate/100,000 population		8,823 (7,753 to 9,893)
Independent variables: demographic		-
Population density (population/square mile)	-	5,304.7 (4,937.5 to 5,671.8)
% White race	-	58.4 (56.3 to 60.4)
% Black race	-	21.2 (19.2 to 23.3)
% Hispanic/Latino	-	36.0 (33.7 to 38.2)
% Other races/multiracial		5.00 (4.5 to 5.6)
% 65 and older	-	9.4 (8.8 to 9.9)
Independent variables: individual level		
Demographic		
Sex (male)	2,497 (56.8)	
Age (years)	-	52.9 (52.2 to 53.6)
Race/ethnicity		
White	2,167 (49.3)	-
Hispanic/Latino	1,118 (25.4)	-
Black	889 (20.2)	-
Other races/multiracial	221 (5.0)	-
Mechanism of injury/hospital records		
Blunt	2,599 (59.4)	-
Penetrating	288 (6.6)	-
Other/unknown	1,608 (34.3)	-
Elixhauser Comorbidity Score	-	Median, 2; range, 0 to 12
Alcohol intoxication at hospital	450 (10.2)	-
Length of hospital stay (days)	-	5.8 (5.5 to 6.0)

+, means are presented unless otherwise specified.

**Table 2:** **Types and frequencies of 17,550 injuries in 6,032 patients hospitalized for traumatic injuries in Dallas County, 2010**

Injuries	Number	Percent
Femur, knee, tibia-fibula, ankle, foot fractures	3,224	18.37
Arm, elbow, wrist, or hand fracture/dislocations	1,526	8.70
Brain injuries +/− coma	1,455	8.29
Open wound injuries	1,227	6.99
Eye, ear, face soft tissue, mouth, teeth injuries	1,066	6.07
Facial fractures	893	5.09
Ill-defined or non-specific injury ICD-9 code	875	4.99
Thorax, heart, lungs, or diaphragm injuries	846	4.82
Pelvic fractures	839	4.78
Rib or sternum fractures	701	3.99
Skull fracture +/− brain injuries	554	3.16
Contusion injuries	513	2.92
Lumbar spine fracture/dislocation +/− spinal cord injuries	504	2.87
Cervical spine fracture/dislocation +/− spinal cord injuries	454	2.59
Pancreas, liver, spleen, adrenal gland injuries	395	2.25
Thoracic spine fracture/dislocation +/− spinal cord injuries	350	1.99
Sprains	343	1.95
Esophagus, stomach, small intestine, colon, rectum injuries	274	1.56
Vascular injuries	244	1.39
Clavicle or scapula fractures	230	1.31
Joint dislocations	217	1.24
Spinal cord or nerve injuries	191	1.09
Sacral/coccyx spine fracture/dislocations	163	0.93
Kidneys, ureter, bladder, urethra injuries	141	0.80
Injuries from foreign bodies	88	0.50
Amputation injuries	76	0.43
Crush injuries	59	0.34
Genitalia, reproductive organ injuries	47	0.27
Burn injuries	34	0.19
Larynx, trachea, or thyroid injuries	21	0.12
Total	17,550	100.00

**Table 3:** **OLS regression model predicting hospitalization for traumatic injury: The effect of neighborhood context**

	***b***	95% CI	***p*** value
Contextual measures			
Alcohol outlets/100,000 population	0.06	−0.15 to 0.25	0.60
UCR crime rate/100,000	−0.0002	−0.001 to 0.0006	0.60
Population density (population/square mile)	−0.004	−0.008 to −0.001	0.006
% White race	1.57	0.79 to 2.36	<0.001
% Black race	1.77	0.92 to 2.61	<0.001
% Hispanic/Latino	−0.84	−1.87 to 0.19	0.11
% 65 and older	12.31	8.41 to 16.21	<0.001
% Younger than 18 years old	−3.73	−6.59 to −0.88	0.01
% Owner-occupied housing	−75.0	−133.9 to −16.1	0.013
% High school graduates	−2.44	−3.57 to −1.31	<0.001
Constant	505.17	267.88 to 742.45	<0.001

**Table 4:** **Hierarchical OLS regression model of individual- and neighborhood-level predictors of injury severity**

	***b***	95% CI	***p*** value
Contextual measures			
Crime rate/100,000 population	2.6 × 10^−6^	−3.9 × 10^−6^ to 9.0 × 10^−6^	0.44
Alcohol outlet density	7.6 × 10^−5^	−0.0009 to 0.001	0.88
Traumas/100,000 population	0.0008	0.0003 to 0.0012	0.002
Patient-level measures			
Elixhauser Comorbidity Score	0.08	0.04 to 0.11	<0.001
Alcohol intoxication at hospital	0.42	0.21 to 0.63	<0.001
Penetrating mechanism of injury	0.38	0.09 to 0.67	0.01
Sex (male)	0.37	0.23 to 0.49	<0.001
White race	−0.28	^-^0.56 to 0.004	0.05
Black race	−0.37	−0.68 to −0.07	0.02
Hispanic/Latino ethnicity	−0.23	−0.52 to 0.06	0.12
Constant	−4.69	−4.87 to −4.51	<0.001

First, we expected that crime rates and alcohol outlet density, independent of SES, patient behavior, and demographics, would be associated with the *incidence* of traumatic injuries sufficient to warrant hospitalization. The mean incidence of hospitalization for trauma was 264.05/100,000 (95% confidence interval (CI) 260/100,000 to 273.83/100,000). Low proportions of owner-occupied housing, followed by the proportion of residents aged 65 years and older, were closely associated with traumatic injury (Table 5). Further, a greater proportion of tract residents younger than 18 was associated with a lower incidence of traumatic injury. A greater proportion of whites and blacks (compared to ‘other races’ and Hispanics/Latinos) living in the neighborhood was also associated with increased trauma rates, as the proportion of white and black residents was positively associated with traumatic injury. Population density (persons per square mile) was modestly protective from trauma, and neither the crime rate nor alcohol outlet densities were significant predictors of the incidence of hospitalization for treatment of traumatic injuries (Table 5).

**Table 5:** **Hierarchical logistic regression analysis predicting** ***mortality*** **among 6,027 trauma patients from Dallas County, Texas**

	OR	95% CI	***p*** value
Contextual measures			
Part One crimes/100,000	1.00	1.00 to 1.00	0.42
Alcohol outlet density	1.00	1.00 to 1.00	0.13
Unemployment rate	1.05	1.00 to 1.09	0.02
Patient-level measures			
Age	1.04	1.01 to 1.09	<0.001
Penetrating mechanism of injury	1.98	1.12 to 3.40	0.02
Severity of injury^a^	2.94	2.55 to 3.40	<0.001
Constant	0.05	0.02 to 0.11	<0.001

This model estimates random effects at the hospital and census tract level. Area under the ROC curve = 0.89, indicating a high sensitivity of the model. ^a^logit transformation of TMPM p(death).

Next, we examined whether neighborhood characteristics, beyond traditional SES measures, would predict the *severity* of traumatic injury and hospital *mortality* as a result of traumatic injury (in particular, the crime rate and density of alcohol retail outlets). We found that neither the rate of Part One crimes nor the density of alcohol retail outlets were associated with greater injury severity (Table 6). Individual characteristics, specifically, the number of medical comorbidities (Elixhauser Comorbidity Score), alcohol intoxication at hospital presentation, penetrating injury, and male sex were all positively associated with the *severity* of the traumatic injury. Black race, however, was associated with less severe injury than other races.

**Table 6:** ***T*** **test or** ***x*** ^**2**^ **comparison of patients included (versus those excluded) and census tracts in Dallas County**

	Included ***N*** (%) or mean (95% CI)	Excluded ***N*** (%) or mean (95% CI)	***p*** value
Patient-level variables	*n* = 6,078	*n* = 161	
Demographic information			
Age	54.6 (54.0 to 55.2)	57.1 (53.7 to 60.6)	0.16
Sex (male)	3,339 (54.8)	78 (48.5)	0.11
Race/ethnicity			0.51
White	3,099 (51.0)	89 (55.3)	
Black	1,216 (20.0)	34 (21.1)	
Hispanic/Latino	1,459 (24.0)	32 (19.9)	
All other races	304 (5.0)	6 (3.7)	
Healthcare/event data			
Uninsured	1,618 (26.6)	25 (15.5)	0.002
Alcohol intoxication	595 (9.8)	13 (8.1)	0.47
Elixhauser Comorbidity Score	2.0 (2.0 to 2.1)	2.1 (1.9 to 2.4)	0.50
Length of hospital stay (days)	5.6 (5.4 to 5.8)	4.6 (3.8 to 5.4)	0.02
Trauma center admission	3,920 (64.5)	95 (59.0)	0.15
Penetrating mechanism of injury	546 (10.8)	8 (5.8)	0.06
TMPM, p(death)	0.05 (0.05 to 0.05)	0.04 (0.03 to 0.07)	0.14
Died	191 (3.2)	3 (1.9)	0.35
Census tract-level variables	*n* = 510	*n* = 16	
Trauma/100,000	344.8 (328.2 to 361.4)	275.5 (220.1 to 331.0)	0.02
Alcohol retail/100,000	49.4 (41.6 to 57.2)	41.7 (18.9 to 64.5)	0.51
Population density	5,311.7 (4,944.2 to 5679.2)	4,146.5 (2,610.6 to 5,682.5)	0.14
% Younger than 18	26.4 (25.7 to 27.1)	27.9 (24.4 to 31.4)	0.38
% 65 and older	9.4 (8.8 to 9.9)	7.9 (6.1 to 9.7)	0.12
% White race	58.5 (56.4 to 60.5)	62.7 (50.1 to 75.2)	0.49
% Black race	21.2 (19.1 to 23.2)	23.5 (9.3 to 37.7)	0.74
% Hispanic/Latino	35.8 (33.5 to 38.0)	36.7 (22.9 to 50.5)	0.89
% High school graduates	76.1 (74.5 to 77.7)	73.5 (62.2 to 84.7)	0.64
% Owner-occupied housing	54.3 (51.9 to 56.6)	60.7 (47.1 to 74.3)	0.34
Median household income in thousands	$55.9 (52.9 to 58.9)	$53.5 (45.2 to 61.8)	0.58
Living below poverty level	18.6 (17.4 to 19.7)	14.1 (9.3 to 21.0)	0.24
Unemployment, age 20 to 64 years old	7.7 (7.3 to 8.1)	6.0 (4.7 to 7.2)	0.01

With respect to *mortality* from traumatic injuries, the unemployment rate was the only contextual SES predictor associated with mortality due to traumatic injury. Neither the crime rate nor the alcohol outlet density of the neighborhood was associated with traumatic mortality among citizens of Dallas County (Table 7). At the patient level, penetrating mechanism of injury was associated with two times the odds of mortality compared to other mechanisms of injury. Finally, more severe injuries were associated with greater mortality, and age was associated with a 4% increase in mortality due to trauma.

**Table 7:** **Logistic regression assessing selection effects for census tracts included in this study**

	Odds ratio	95% CI	***p*** value
Census tract measures			
Trauma/100,000 population	1.00	1.00 to 1.01	0.04
Alcohol outlet density	1.00	1.00 to 1.01	0.66
Demographic measures			
% White race	0.92	0.86 to 0.99	0.02
% Black race	0.92	0.86 to 0.98	0.01
% Other race	Ref	-	
High school graduates	1.07	1.01 to 1.11	0.001
Below poverty level (past 12 months)	1.10	1.03 to 1.17	0.004
Constant	12.45	0.07 to 2,213.96	0.34

## 4 Discussion

We found that several well-established neighborhood-level SES measures were significantly associated with injury that required hospital admission and treatment. However, as expected, these predictors were less robust than individual-level characteristics of the patient. Further, the indicators of alcohol outlet density and crime rate in the neighborhood were not predictors in the present analysis. In summary, we found that other neighborhood-level SES predictors of traumatic injury included a lower population density, lower proportion of owner-occupied housing (e.g., more rental properties in a given neighborhood), the proportion of the tract population 18 years old or younger, as well as the percentage 65 years and older. Interestingly, both the neighborhood proportions of whites and black/African Americans exhibited a modest but significant increase in the incidence of hospitalization to treat traumatic injuries.

The relationship between traumatic injury and neighborhood characteristics has been studied from various perspectives. Zarzur et al. found that neighborhood SES was inversely related to incidence rates of trauma; however, SES was defined only in terms of neighborhood income cut points in one county in Tennessee (Zarzur et al. [2010]). Cubbin and colleagues noted that SES (in this case, income-to-need ratio, education level, occupation, race, ethnicity) is a robust predictor of mortality due to traumatic injury; however, the effect depended upon the indicator of SES and the cause and severity of injury (Cubbin and Smith [2002]). Such factors include risk-taking behavior, comorbid conditions, alcohol intoxication, and mechanism of injury. Since these phenomena appear to be multifactorial in nature, they should be investigated in a similar fashion. As we sought to understand how each measure of SES is *independently* associated with trauma morbidity and mortality, multiple levels of data (e.g., both neighborhood and individual level measures of income and poverty) were applied.

Injury prevention is a matter of increasing significance, as the Institute of Medicine published the report, ‘Hospital-Based Emergency Care: At the Breaking Point’ due to the increasing demands of emergency and trauma care and the current capacity limitations in 2007 (Committee on the Future of Emergency Care in the United States Health System [2007]). The authors state that, ‘In 2003, nearly 114 million visits were made to hospital emergency departments… About one-quarter of those visits were due to unintentional injuries.’ In 2011, the American Association for the Surgery of Trauma Prevention Committee issued a call to action for trauma centers, which recommended that prevention programs target neighborhoods where important socioeconomic and cultural factors need to be identified (Davis et al. [2011]).

Although the relationship between race and traumatic injury has been well documented, there is limited research that incorporates both individual race of the patient and the racial composition of their home neighborhood. For instance, at the individual level, Cubbin et al. ([2000]) found that black adults were at 61% age-adjusted greater risk for traumatic injury compared to whites using the National Health Interview Survey data. Newgard and colleagues conducted an ecological study of emergency medical system (EMS) data from nine cities across the USA and Canada. This study reported a positive association between the rate of major traumatic injuries and the percentage of the non-white population (Newgard et al. [2011]). Our results were concordant with the literature on race and traumatic injury, as we found a positive relationship between racial composition at the neighborhood level (e.g., proportion of black and white), and traumatic injury in general. However, we did find that blacks and whites (compared with Hispanic/Latinos and other racial groups) were more likely to have injuries of greater severity. These studies were substantively different from the present investigation, as each study evaluated one level of data only, and Cubbin and colleagues assessed mortality only, with risk factors assessed only at the individual level.

The role of ethnicity at the individual and neighborhood level provides an interesting albeit complex and multidimensional research question as it relates to traumatic injury and neighborhood context. Hispanics have rates of violence and crime that are closely approaching and surpassing those of blacks, and they are likely to live in areas that are characterized by social disorder, poor living conditions, and poverty (Gonzalez-Barrera and Lopez [2013]; Reingle et al. [2012]). Therefore, given the potential for traumatic injury due to violence and crime-related activity, we were surprised that Hispanic ethnicity was not identified as a risk factor for injury, injury severity, or mortality. We can only postulate that this may be a function of a lower likelihood of treatment seeking or measurement error (for instance, those of ‘Hispanic/Latino’ descent may be less likely to self-report this as a personal identity). However, elucidating the phenomena underlying this finding is beyond the scope of this study.

Our results regarding alcohol outlet density and trauma differ from the extant literature, and we expect that methodological differences may explain much of this variability. For instance, Mair et al. investigated the association between alcohol retail density, specifically bars, and assault-related injury hospitalizations. Mair observed a positive relationship between the number of alcohol outlets and injury due to assault; however, this effect varied according to the demographic characteristics of the neighborhood (Mair et al. [2012]). In addition, studies of alcohol policy have found that reducing alcohol availability by reducing the number of alcohol outlets in a community or the hours and days of sale resulted in lower rates of injury almost immediately (Zhu et al. [2004]; Scribner et al. [1999]; Britt et al. [2005]; Popova et al. [2009]). Therefore, we expect that this differential finding is a function of our inclusion of both on- and off-premise alcohol retail outlets as this distinction has been shown to have unique effects on outcome (Campbell et al. [2009]). Further, alcohol retail outlets are more densely located in lower socioeconomic locations (Berke et al. [2010]). Although not the primary focus of this paper, we believe that the intrinsic correlation between alcohol outlets and neighborhood SES will account for the lack of observed independent effect of alcohol outlets on traumatic injury.

Mortality from traumatic injury has long been the focus of research to identify risk and protective factors that are amendable to intervention in the hospital setting. Typically, this research models the risk of highly *specific* types of injuries, injury patterns, or the benefits of various treatments. However, researchers have recently begun to expand the scope of their research beyond the hospital walls to investigate how features of the geographic and social environment contribute to traumatic injury mortality (Alkhoury et al. [2009]; Arthur et al. [2008]; Centerwall [1995]; Chapman et al. [2010]; Cubbin et al. [2000]; Haider et al. [2008]). This inclusion of administrative, diagnostic, trauma registry data has tremendous potential to identify the cases of injury and mortality where self-reported information and arrest data do not suffice.

Our study has several limitations. First, we limited our geographic scope to include the census tracts with crime rate data (excluding six police jurisdictions that did not provide such data). While this allowed inclusion of 89% of the land area and 97.1% of the county population, limited selection bias was observed (see Tables 2, 3, and 4). Other limitations of this study include the administrative nature of the hospital discharge data. Although 64.2% of the patients in this study were treated at verified trauma centers, where registrars carefully document pertinent injury and clinical data, no clinical or administrative database is entirely free of error. Next, our analyses treat the patient as if all received comparable levels of exposure to the neighborhood characteristics (at the census tract level) examined, and the duration of each patient's exposure to their respective environments cannot be measured with these data. Moreover, all traumatic injuries were given equal weight with regard to association with the predictors though differences in association may exist among subgroups of injuries and predictors. Finally, all of the UCR index crimes were aggregated to define a tract's ‘crime rate,’ as we did not have a specific hypothesis as to which types of crime might be most closely related to traumatic injury.

In light of these limitations, the major strength of this paper is the broad, epidemiologic perspective it provides. Despite the large number of studies that have been done in this field, this study is one of the most comprehensive*,* investigatory, epidemiological studies to date to establish the correlates at both the neighborhood and individual level using hospital data, census information, police records, and alcoholic beverage licensing datasets. Further, we were able to establish direct associations between multi-level measures and ‘injury’ in general. This is important epidemiologically in terms of prevention; communities with high crime rates or diverse racial compositions may be identified as ideal locations for primary prevention.

## 5 Conclusions

Although individual-level measures were more robust predictors of traumatic injury and mortality, neighborhood-level crime and context plays a role in the incidence of traumatic injury requiring hospitalization. Further research is needed to assess which injury prevention efforts may yield the greatest benefit in reducing rates of traumatic injury and mortality, as even small effects at the neighborhood level may have vast effects on the targeted population as a whole.

## Authors’ original submitted files for images

Below are the links to the authors’ original submitted files for images. Authors’ original file for figure 1 Authors’ original file for figure 2 Authors’ original file for figure 3

## Abbreviations

95% CI: 95% confidence interval
DFWHCF: Dallas-Fort Worth Hospital Council Foundation
ICD-9: International Classification of Diseases, Ninth Edition - Clinical Modification
ROC: receiver operator characteristic
SES: socioeconomic status
UCR: Uniform Crime Report
