# Supplementary figures and images for: Do neighborhood demographics, crime rates, and alcohol outlet density predict incidence, severity, and outcome of hospitalization for traumatic injury? A cross-sectional study of Dallas County, Texas, 2010
Source: Inj Epidemiol. 2014 Oct 20;1(1):23. doi: 10.1186/s40621-014-0023-2 (PMC5005658; doi:10.1186/s40621-014-0023-2)

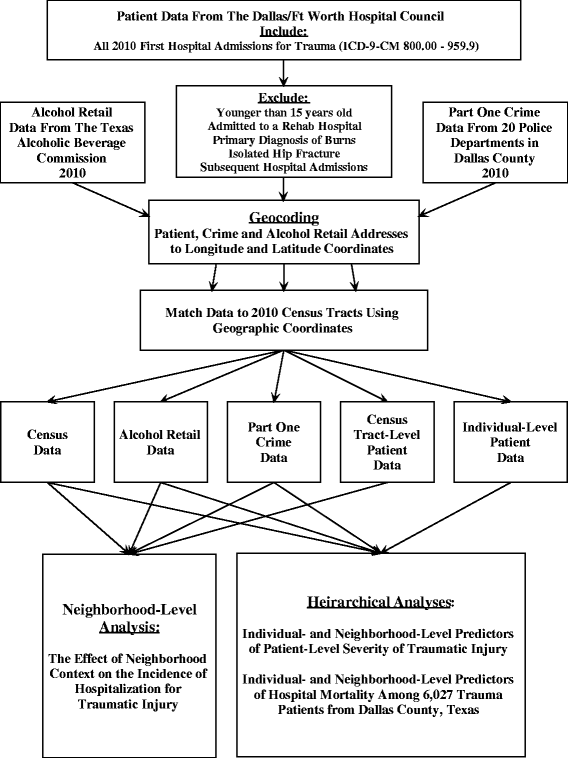

Supplement: Supplementary file 1 — Authors’ original file for figure 1 [file 40621_2014_23_MOESM1_ESM.gif]

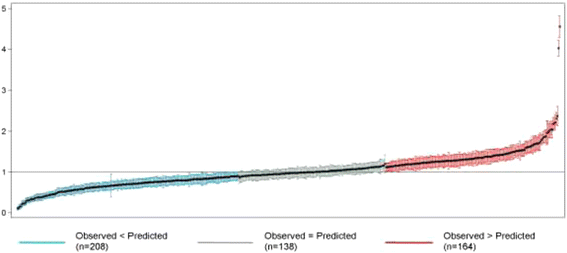

Supplement: Supplementary file 2 — Authors’ original file for figure 2 [file 40621_2014_23_MOESM2_ESM.gif]

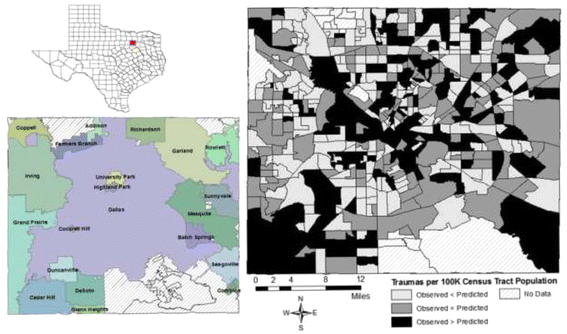

Supplement: Supplementary file 3 — Authors’ original file for figure 3 [file 40621_2014_23_MOESM3_ESM.gif]
